# Metformin Increases Sensitivity of Melanoma Cells to Cisplatin by Blocking Exosomal-Mediated miR-34a Secretion

**DOI:** 10.1155/2021/5525231

**Published:** 2021-11-29

**Authors:** Lan Ge, Yaguang Wu, Ming Wan, Yi You, Zhifang Zhai, Zhiqiang Song

**Affiliations:** ^1^Department of Dermatology, Southwest Hospital, Third Military Medical University, Chongqing 400038, China; ^2^Shumei Cosmetic Clinic in Lianhu District, Xi'an 710075, China

## Abstract

Melanoma, also known as malignant melanoma, is a type of cancer derived from the pigment-containing cells known as melanocytes. Cisplatin (CDDP) is widely used in the treatment of different types of tumors with high response rates, but it generally has low efficiency in melanoma. This study aimed to investigate whether metformin could sensitize the melanoma cell line A375 to cisplatin. Our results for the first time indicated that CDDP increased the miR-34a secretion by exosomes in melanoma A375 cells, which was, at least partially, related to the cisplatin resistance of melanoma cells. Moreover, metformin significantly sensitized A375 cells to cisplatin. Mechanistically, metformin significantly blocked the exosome-mediated miR-34a secretion induced by cisplatin. Our study not only reveals a novel mechanism that exosomal secretion of miR-34a is involved in the cisplatin resistance of melanoma cells but also provides a promising therapeutic strategy by synergistic addition of metformin.

## 1. Introduction

Melanoma is one of the most aggressive cancer, and its incidence is still increasing almost all over the world [1]. It has been well established that BRAF^V600E^ is the most common mutation type, occurring in about 50% of melanoma patients. BRAF^V600E^ mutation results in hyperactivation of the MAPK pathway and subsequent uncontrolled cell growth. The traditional chemotherapeutic drugs, such as cisplatin (CDDP, cis-diaminodichloroplatinum), generally has a low efficiency for melanoma [2]. Although cisplatin is one of the most commonly used chemotherapeutic agents in multiple solid cancers [3], it has been confirmed that melanoma is inherently resistant to CDDP, but the mechanisms are not fully understood. Unraveling the underlying mechanism by which melanoma is inheritably resistant to cisplatin would be helpful for the development of therapeutic strategy for melanoma. In a recent study, Sun et al. revealed that CDDP-induced senescence-associated secretory factors were involved in the drug resistance [2].

Besides the routine secretory factors, exosomes are recognized as pivotal mediators of cell-cell communication through transferring genetic cargos such as miRNAs and other noncoding RNAs [4]. For example, cancer cell-derived exosomes are found to encapsulate noncoding RNAs in the donor cells, and the exosomes in turn are endocytosed by the recipient cells, which promote cancer development [5]. Recently, Gao et al. found that CML cells could promote cell growth via exosomal sorting of tumor suppressive miRNAs (mainly miR-320) [6], suggesting that exosomal secretion of tumor suppressive miRNA might be a common event in cancer development and drug resistance. Since 2007, reports from several laboratories have shown that the members of miR-34 family (namely, miR-34a, miR-34b, and miR-34c) are direct p53 targets, and their upregulation induces apoptosis and cell-cycle arrest [7–10]. In mammalians, miR-34a is encoded by its own transcript, whereas miR-34b and miR-34c share a common primary transcript. A previous study in mice revealed that miR-34a is ubiquitously expressed, whereas miR-34b/c is mainly expressed in lung tissues [11]. It is thus interesting to test whether miR-34a can be secreted via the exosomes in the cisplatin-treated melanoma cells.

Metformin has been widely used for the treatment of type 2 diabetes and prediabetic conditions for half a century due to its good tolerability and low cost. Recently, epidemiological studies and basic research studies have suggested that metformin may reduce the risk of cancer in diabetic patients and prolong the survival rate of cancer patients [12–14]. These results suggest that metformin may potentially be used as an anticancer adjuvant for the treatment of some cancers. However, whether metformin can be used in melanoma therapy remains unknown, not even to the possible mechanisms of action.

In this study, the exosomal secretion of miR-34a was investigated in cisplatin-treated melanoma, and whether metformin could sensitize the melanoma cells to cisplatin and the role of exosomal secretion of miR-34a were further explored.

## 2. Materials and Methods

### 2.1. Cell Culture and Reagents

Melanoma cell line A375 was purchased from the Type Culture Collection of the Chinese Academy of Sciences, Shanghai, China. Cells were cultured in DMEM containing 10% fetal bovine serum (FBS) and 1% antibiotics (Hyclone, USA). CDDP (P4394) and metformin (BP227, metformin hydrochloride) were from Sigma-Aldrich Co., USA.

### 2.2. Exosome Purification

Before exosome isolation from A375 cells with or without cisplatin treatment, cells were cultured in 10% exosome-depleted FBS (ultracentrifugation at 120,000 g for 16 h). Exosomes were isolated by differential centrifugation. Briefly, the culture supernatant was collected after 48–72 h culture, followed by centrifuged at 800 g for 5 min and 2000 g for 10 min. The supernatant was then filtered via a 0.22 *μ*m filter, followed by centrifugation at 10,0000 g for 2 h. The pellet was resuspended in PBS. The concentration and size distribution of exosomes were then determined.

### 2.3. Quantitative Real-Time PCR

Total RNA was extracted from cells or exosomes with TRIzol (Invitrogen) following the manufacturer's instructions. RNA purity and concentration were assessed with a Nanodrop-1000 spectrophotometer. miRNA reverse transcription was performed using the miRcute miRNA First-Strand cDNA Synthesis Kit. Relative expression was then analyzed by qPCR using the miRcute miRNA SYBR Green qPCR Detection Kit (Tiangen, Beijing, China). U6 was used as an internal reference. The relative expression of miRNA was calculated by the 2^−ddCt^ method. The forward primers used were as follows: miR-34a, 5′-TGGCAGTGTCTTAGCTGGTTGT-3′; U6, 5′-GGATGACACGCAAATTCGTGAAGC-3′. The reverse primer was provided in the kit.

### 2.4. Western Blotting

Cells were lysed in the lysis buffer (20 mM Tris (pH 7.5), 150 mM NaCl, 1% Triton X-100 2 mM sodium pyrophosphate, and 25 mM *β*-glycerophosphate) supplemented with protease inhibitor cocktail (Roche). Protein extracts were separated in 12% SDS-PAGE and transferred onto nitrocellulose membrane. The nitrocellulose membrane was blocked with 5% nonfat milk for 1 h and then incubated overnight with primary antibodies (anticleaved caspase-3 antibody (ab2302), 1 : 500; anti-*β*-actin antibody (ab2302) and anti-*β*-actin antibody (ab8227), 1 : 2500) at 4^o^C. The membrane was then incubated for 1 h with peroxidase-conjugated secondary antibodies at room temperature and developed using the ECL Prime Western Blotting Detection Reagent.

### 2.5. Cell Counting Kit-8 Assay

Cell proliferation was analyzed using the Cell Counting Kit-8 (CCK-8) (96992, Sigma-Aldrich). Briefly, A375 cells with metformin/cisplatin treatment or the control cells were seeded in 96-well plates at 800 cells/well. The cell survival was monitored by adding 10 *μ*l of CCK-8 reagent at predesigned time points, followed by additional incubation for another 2 h. Absorbance was measured at 450 nm in a microplate reader.

### 2.6. Statistical Analysis

Data were analyzed with GraphPad Prism7 software. The unpaired two-tailed *t*-test was used to compare data between two groups. The two-way or one-way ANOVA test was used to compare data among groups. Data are presented as the mean ± standard error (SEM). A value of *P* < 0.05 was considered statistically significant.

## 3. Results

### 3.1. Melanoma Cells Were Intrinsically Resistant to Cisplatin due to Reduced miR-34a Expression

Cisplatin treatment slightly reduced the proliferation rate of A375 cells as shown by both CCK-8 assay (Figure S1(a)) and slightly increased the production of cleaved caspase-3 as shown by Western blotting (Figure S1(b)).

**Figure 1:**
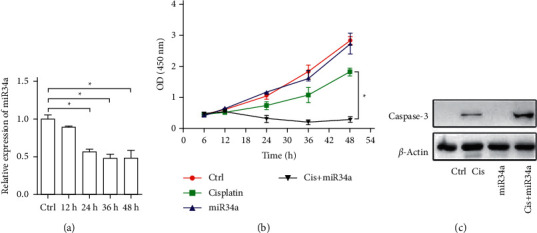
Sensitivity of miR-34a A375 cells to cisplatin. qPCR of miR-34a expression in A375 cells at different time points after cisplatin treatment. U6 served as internal control. ^*∗*^*P* < 0.05 (one-way ANOVA). Cell survival of A375 cells (CCK-8 assay). (b) Cells transfected with negative control (NC) or miR-34a further treated with vehicle or cisplatin, followed by CCK-8 assay at indicated time points. ^*∗*^*P* < 0.05 (two-way ANOVA). (c) Western blotting of cleaved caspase-3 in A375 cells. Cells treated as abovementioned were harvested at indicated time points. *β*-Actin served as an internal reference. Data are representatives of 3 independent experiments.

To further explore the underlying mechanism by which A375 cells were insensitive to cisplatin, the role of miR-34a was further investigated because miR-34a was a tumor suppressive miRNA regulated by p53. Unexpectedly, miR-34a expression was downregulated in a time-dependent manner, rather than upregulated upon cisplatin treatment in the A375 cells (Figure 1(a)). Transfection of miR-34a significantly sensitized A375 cells to cisplatin as shown by the increased apoptosis (Figures 1(b) and 1(c)).

### 3.2. Exosomal Secretion of miR-34a Contributes to the Cisplatin Insensitivity

**Figure 2:**
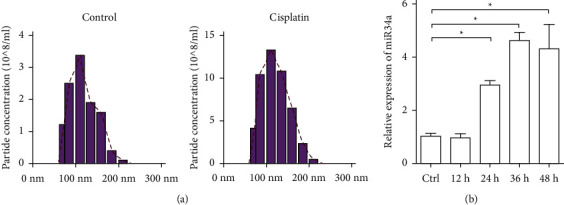
Role of exosome secretion of miR-34a in the sensitivity of A375 cells to cisplatin. Exosome size distribution and concentration analysis. Exosomes from cells treated with or without cisplatin were analyzed by NanoSight. Data are representative of 3 independent experiments. (b) qPCR of miR-34a expression in the exosomes from A375 cells at different time points after cisplatin treatment. ^*∗*^*P* < 0.05 (one-way ANOVA).

We hypothesized that exosome secretion promoted miR-34a secretion upon cisplatin treatment. As shown in Figure 2(a), cisplatin treatment significantly increased the exosome secretion, while the size of exosomes remained unchanged. Consistent with the increased exosome secretion, the miR-34a in the exosomes was also found to be significantly enriched (Figure 2(b)).

**Figure 3:**
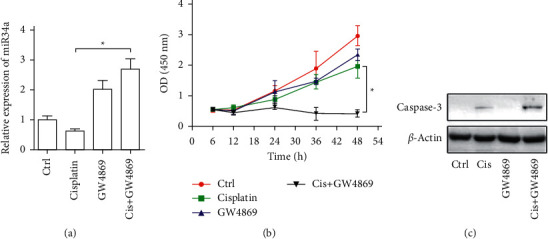
Exosome secretion blockade sensitizes A375 cells to cisplatin. qPCR of miR-34a expression in A375 cells. Cells were treated with exosome secretion inhibitor GW4869, followed by control or cisplatin treatment. (b) Survival of melanoma cell (A375 cells) (CCK-8 assay). Cells were treated with exosome secretion inhibitor GW4869, followed by control or cisplatin treatment. ^*∗*^*P* < 0.05 (two-way ANOVA). (c) Western blotting of cleaved caspase-3 in A375 cells. Cells treated as abovementioned were harvested at indicated time points. *β*-Actin served as internal reference. Data are representatives of 3 independent experiments.

Moreover, in the presence of cisplatin, the addition of GW4869 to inhibit exosome secretion significantly increased the endogenous expression of miR-34a in the A375 cells (Figure 3(a)), reduced cell death, but increased the apoptosis in the A375 cells (Figures 3(b) and 3(c)).

### 3.3. Metformin Treatment Sensitizes A375 Cells to Cisplatin via Blocking Exosomal Secretion of miR-34a

**Figure 4:**
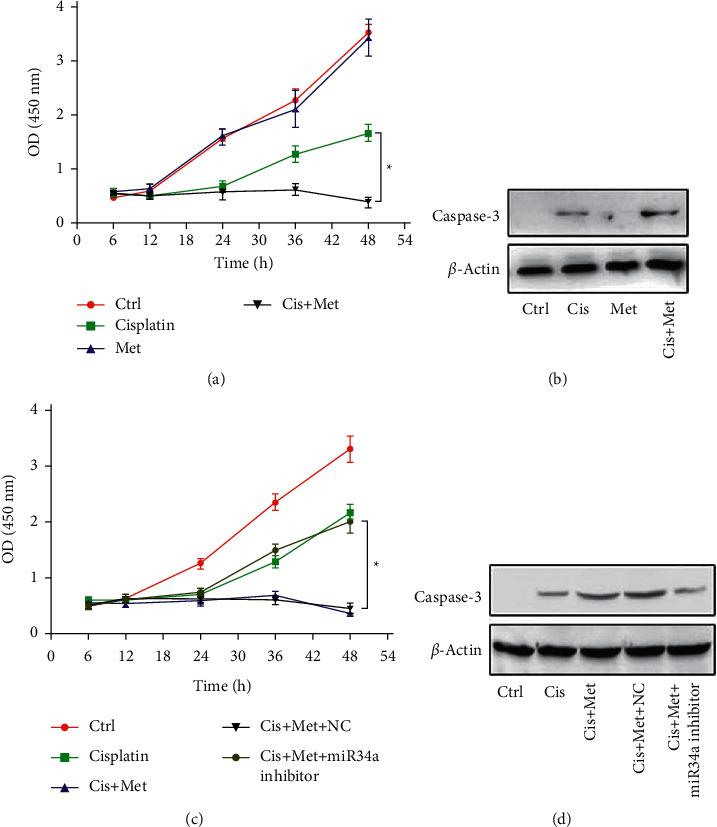
Metformin treatment sensitizes A375 cells to cisplatin via blocking exosomal secretion of miR-34a. Survival of melanoma cell A375 cells (CCK-8 assay). Cells were treated with metformin, followed by control or cisplatin treatment. ^*∗*^*P* < 0.05 (two-way ANOVA). (b) Western blotting of cleaved caspase-3 in A375 cells. Cells treated as abovementioned were harvested at indicated time points. *β*-Actin served as internal reference. Data were representative of 3 independent experiments. (c) Survival of melanoma cell (A375 cells) (CCK-8 assay). Cells transfected with NC or miR-34a inhibitor were additionally treated with control or metformin/cisplatin treatment. ^*∗*^*P* < 0.05 (two-way ANOVA). (d) Western blotting of cleaved caspase-3 in A375 cells. Cells transfected with NC or miR-34a inhibitor were additionally treated with control or metformin/cisplatin treatment. *β*-Actin served as internal reference. Data are representatives of 3 independent experiments.

Metformin is recently found to increase the sensitivity of cancer cells to chemotherapy. Then, the role of metformin in sensitizing A375 cell to cisplatin was further investigated. As expected, metformin significantly promoted the cisplatin-induced cell death (Figures 4(a) and 4(b)). Moreover, metformin significantly decreased the exosome secretion induced by cisplatin (Figure S2(a)), together with the increased endogenous expression of miR-34a (Figure S2(b)). Notably, metformin alone had mild effect on the miR-34a expression, while cisplatin plus metformin markedly enhanced the miR-34a expression. To further confirm whether metformin sensitized cells via blocking exosomal miR-34a secretion, miR-34a expression was simultaneously knocked down in A375 cells. Results showed that knockdown of miR-34a expression almost totally blocked the effects of metformin (Figures 4(c) and 4(d)). These findings indicate that metformin sensitizes A375 cells to cisplatin-induced death via blocking exosomal secretion of miR-34a.

## 4. Discussion

Melanoma is an incredibly virulent disease, but its diagnosis and treatment are difficult because it is a heterogeneous and complex disease. Currently, the primary treatment for localized melanoma is the surgical removal of the tumor and surrounding healthy tissue, and sentinel lymph node biopsy is usually performed in high-risk patients [15]. However, surgical treatment alone will not be curative for patients with metastatic disease, and thus, pharmacotherapies are the next line of treatment. To date, chemotherapy is the only treatment option for patients with metastatic melanoma.

CDDP-based treatment is still a classic treatment for metastatic melanoma in most cases, and it is frequently used in combination with other therapies [16]. However, the resistance of melanoma cells to CDDP-based drugs has been a challenge in the clinical treatment of melanoma. Currently, the mechanisms underlying the resistance of melanoma to chemotherapy are still poorly understood. Previously, cisplatin was found to induce DNA double-strand breaks via Pt-DNA adducts, and thus, the cells died if the DNA breaks were not fully repaired by nucleotide excision repair, nonhomologous end joining (NHEJ), or homologous recombination (HR) [17, 18]. In a recent study, Weng et al. found that cisplatin could induce giant cells formation, which was involved in the chemoresistance of melanoma cells [19]. A study of Zhuo et al. revealed that hypoxia potentiated the capacity of melanoma cells to evade cisplatin [20]. Karwaciak et al. found SIRT2 inhibition could increase the sensitivity to cisplatin [21].

In recent years, increasing studies have revealed that aberrant miRNAs frequently are involved in the pathogenesis of various types of cancers through regulating many cancer-pertinent cellular processes, including chemoresistance, and deregulation of microRNAs and their targeted genes in melanoma development with the hallmarks and characteristics of cancer. Li et al. found silencing of microRNA-211 was able to decrease the sensitivity of melanoma cells to cisplatin [22]. A study of An et al. revealed that downregulation of lncRNA H19 was able to sensitize melanoma cells to cisplatin by regulating the miR-18b/IGF1 axis [23]. In previous studies, miR-34a was characterized as a tumor suppressor [24], and miR-34 dysregulation may be involved in the development of some cancers. The miR-34a expression was lower in metastatic melanoma cell lines as compared to in situ melanoma cell line; miR-34a overexpression significantly inhibited WM451 cell proliferation and metastasis, but miR-34a inhibition was found to promote proliferation and metastasis of WM35 [25]. This was confirmed in malignant melanoma with the wild-type p53 gene [26]. Xu et al. found ZEB1 was a target of miR-34a in melanoma cells [27]. It has been revealed that there is a strong association between cisplatin treatment and miR-34a expression in the muscle-invasive bladder cancer and lung cancers [28, 29]. Moreover, cisplatin can induce the expression of miR-34a via different mechanisms, such as epigenetic activation [28], p53 activation, and other transcription factors [30].

In recent years, studies have revealed that exosomes play a vital role in the intercellular communication within the tumor microenvironment, metastasis, and drug resistance [31]. Exosomes are extracellular vesicles and can transport proteins, affect maturation, differentiation, and other processes, and induce genetic change in cells. There is evidence showing that exosomes are relatively stable and resist proteolytic and nuclease activity, and thus, the proteins and nucleic acids enclosed within these vesicles are protected from degradation [32]. In a recent study, results showed human melanoma exosomes could downregulate T cell responses through decreased T cell receptor (TCR) signaling and diminish cytokine and granzyme B secretions; miRNAs enriched in these exosomes, and miRNAs in the melanoma-derived exosomes could assist immune evasion of melanoma and may serve as a therapeutic target [33].

Different from above findings, our results showed that cisplatin decreased miR-34a expression via exosomal secretion. The inconsistence may be explained as the insensitivity of melanoma cell to cisplatin, while bladder and lung cancer cells are sensitive to cisplatin. The mechanism by which miR-34a responds differently in different types of cancer cells remains unclear, and the genetic and environmental differences might be, at least partially, involved in this discrepancy. Thus, more studies are needed to confirm our findings in the future.

Metformin is a well-known inhibitor of the mitochondrial electron transport chain and may cause an abnormal flow of electrons to oxygen, leading to the accumulation of reactive oxygen species (ROS). Studies have linked the antitumor effect of metformin to the generation of ROS [34–36]. In addition, metformin is also an AMPK activator, and AMPK activation has been found to sensitize cancer cells to death via autophagy [37]. In some other studies, metformin activates the expression of proapoptotic protein BAX and decrease the expression of the antiapoptotic protein BCL-2 [38]. Different from above findings, our results showed metformin increased the cancer cell death via promoting the exosomal secretion of tumor suppressive miRNAs.

Recently, exosomes have been found to selectively encapsulate certain miRNAs/mRNAs by RNA binding proteins and thus can directionally regulate the functions of both donor cells and recipient cells [6, 39–41]. For example, heterogeneous nuclear ribonucleoprotein A2B1 and heterogeneous nuclear ribonucleoprotein Q (HNRNPQ) have been found to selectively bind miRNAs containing a certain sequence called EXO motif and guide their entrance into exosomes [39, 40]. In addition, Gao et al. found that HNRNPA1 at least selectively sorted out miR-320 into exosomes via the AGAGGG motif in the miRNAs [6]. Besides the pathophysiological RNA sorting into exosomes, exosomes were engineered by RNA binding protein to load mRNA/miRNA of interest. It is thus interesting to explore the detailed mechanism by which miR-34a is selectively secreted upon cisplatin treatment. It is also reasonable to investigate whether other tumor suppressive miRNAs are also secreted together with miR-34a and whether these tumor suppressive miRNAs, as in miR-34a, in the exosomes also contribute to the resistance of melanoma cells to cisplatin.

## 5. Conclusions

Together, our study for the first time reports that CDDP treatment promotes the exosomal secretion of miR-34a in the melanoma A375 cells, resulting in the resistance to cisplatin-induced apoptosis. In contrast, metformin treatment significantly blocks the exosome-mediated miR-34a secretion following cisplatin treatment and thus sensitizes these cells to cisplatin. Our study not only reveals a novel mechanism that exosomal secretion of miR-34a is related to the resistance of melanoma cells to cisplatin but also provides a promising therapeutic target for the future treatment of melanoma.

## Data Availability

The data used to support the findings of this study are available from the corresponding author upon request.

## Supplementary Materials

Supplementary Materials Figure S1: cisplatin induces minor apoptosis in melanoma cell. (a) Cell survival of melanoma cell (A375 cells) analyzed by CCK-8 assay. ^*∗*^*P* < 0.05 by two-way ANOVA. (B) Western blot analysis of cleaved caspase-3 in A375 cells. Cells with or without cisplatin treatments were harvested at indicated time. *β*-Actin served as internal control. Data shown were representative of 3 independent experiments. Figure S2: metformin inhibits cisplatin-induced exosomal secretion of miR-34a. (a) Exosome size distribution and concentration analysis. Exosomes from cells treated with or without cisplatin/metformin were analyzed by NanoSight. Data are representative of 3 independent experiments. (b) qPCR analysis of miR-34a expression in A375 cells with indicated treatments. U6 served as the internal control. ^*∗*^*P* < 0.05 by one-way ANOVA.
