# Supplementary material for: Metformin Increases Sensitivity of Melanoma Cells to Cisplatin by Blocking Exosomal-Mediated miR-34a Secretion
Source: J Oncol. 2021 Nov 29;2021:5525231. doi: 10.1155/2021/5525231 (PMC8648459; doi:10.1155/2021/5525231)
Supplement: Supplementary Materials — Figure S1: cisplatin induces minor apoptosis in melanoma cell. (a) Cell survival of melanoma cell (A375 cells) analyzed by CCK-8 assay. ∗P < 0.05 by two-way ANOVA. (B) Western blot analysis of cleaved caspase-3 in A375 cells. Cells with or without cisplatin treatments were harvested at indicated time. β-Actin served as internal control. Data shown were representative of 3 independent experiments. Figure S2: metformin inhibits cisplatin-induced exosomal secretion of miR-34a. (a) Exosome size distribution and concentration analysis. Exosomes from cells treated with or without cisplatin/metformin were analyzed by NanoSight. Data are representative of 3 independent experiments. (b) qPCR analysis of miR-34a expression in A375 cells with indicated treatments. U6 served as the internal control. ∗P < 0.05 by one-way ANOVA. [file 5525231.f1.docx]

**Supplementary Information**

**Metformin promotes melanoma cell sensitivity to cisplatin by blocking exosomal mediated miR-34a secretion**

Lan Ge^1#^, Yaguang Wu^1#^, Ming Wan^2^, Yi You^1^, Zhifang Zhai^1^, Zhiqiang Song^1*^

1 Department of Dermatology, Southwest Hospital, Third Military Medical University, Chongqing, 400038, China

2 Shumei Cosmetic Clinic in Lianhu District, Xi’an, 710075, China

# These authors contributed equally to this article.

*Correspondence should be addressed to: songzhiq_cq@163.com


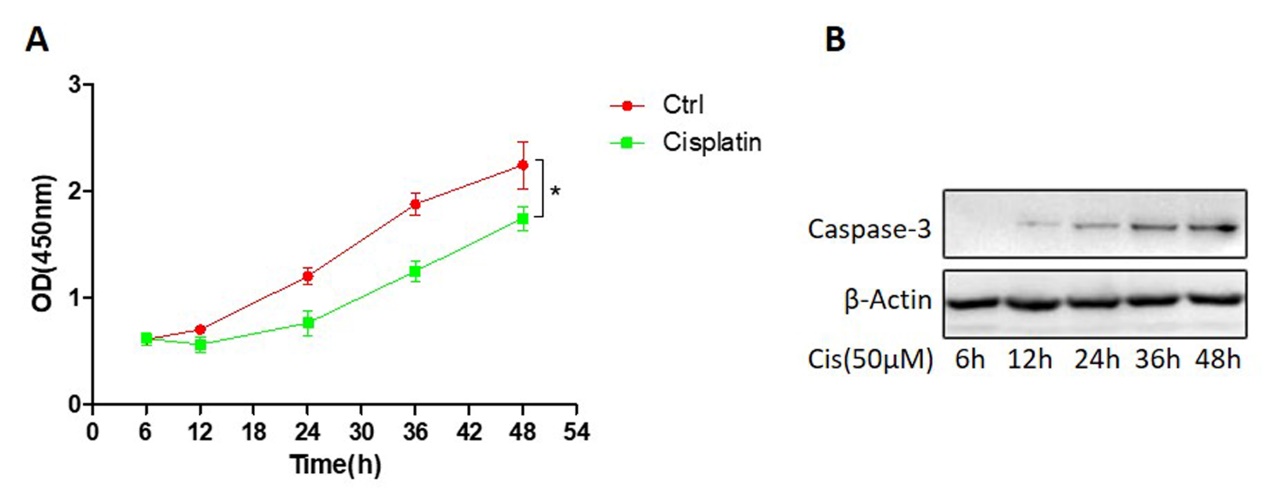


**Figure S1 Cisplatin induces minor apoptosis in Melanoma cell.**

(A) Cell survival of melanoma cell A375 cells analyzed by CCK-8 assay. *,p$<$0.05 by two way ANOVA. (B) Western blot analysis of cleaved caspase-3 in A375 cells. Cells with or without cisplatin treatments were harvested at indicated time. β-actin served as internal control. Data shown were representative of 3 independent experiments.

**
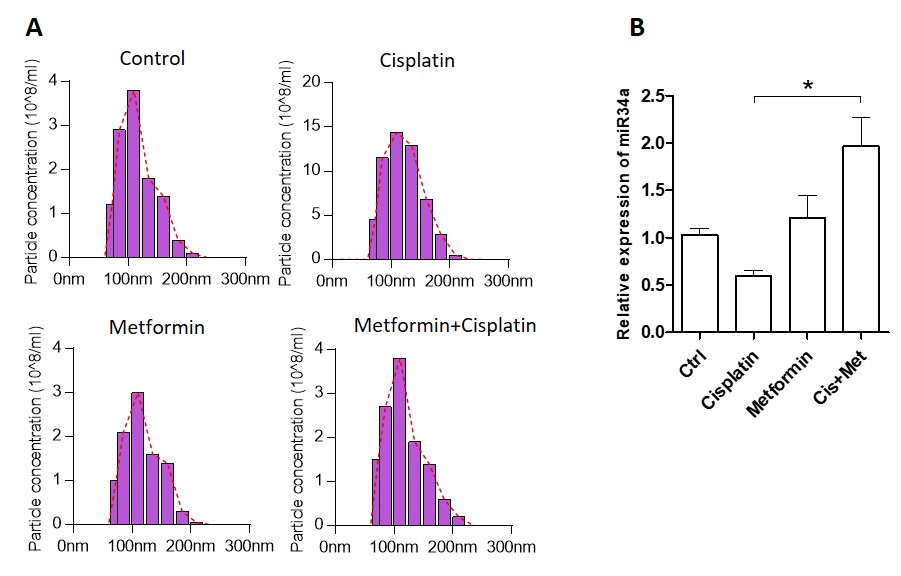
**

**Figure S2 Metformin inhibits cisplatin-induced exosomal secretion of miR-34a.**

(A) Exosome size distribution and concentration analysis. Exosomes from cells treated with or without cisplatin/metformin were analyzed by Nanosight. Data shown were representative of 3 independent experiments. (B) qPCR analysis of miR-34a expression in A375 cells with indicated treatments. U6 served as internal control. *, p$<$0.05 by one way ANOVA.
